# Altered Mitochondrial Function and Energy Metabolism Is Associated with a Radioresistant Phenotype in Oesophageal Adenocarcinoma

**DOI:** 10.1371/journal.pone.0100738

**Published:** 2014-06-26

**Authors:** Niamh Lynam-Lennon, Stephen G. Maher, Aoife Maguire, James Phelan, Cian Muldoon, John V. Reynolds, Jacintha O’Sullivan

**Affiliations:** 1 Department of Surgery, Trinity College Dublin, Dublin, Ireland; 2 Cancer Biology and Therapeutics Lab, School of Biological, Biomedical and Environmental Sciences, University of Hull, Hull, United Kingdom; 3 Department of Pathology, St James’s Hospital, Dublin, Ireland; ENEA, Italy

## Abstract

Neoadjuvant chemoradiation therapy (CRT) is increasingly the standard of care for locally advanced oesophageal cancer. A complete pathological response to CRT is associated with a favourable outcome. Radiation therapy is important for local tumour control, however, radioresistance remains a substantial clinical problem. We hypothesise that alterations in mitochondrial function and energy metabolism are involved in the radioresistance of oesophageal adenocarcinoma (OAC). To investigate this, we used an established isogenic cell line model of radioresistant OAC. Radioresistant cells (OE33 R) demonstrated significantly increased levels of random mitochondrial mutations, which were coupled with alterations in mitochondrial function, size, morphology and gene expression, supporting a role for mitochondrial dysfunction in the radioresistance of this model. OE33 R cells also demonstrated altered bioenergetics, demonstrating significantly increased intracellular ATP levels, which was attributed to enhanced mitochondrial respiration. Radioresistant cells also demonstrated metabolic plasticity, efficiently switching between the glycolysis and oxidative phosphorylation energy metabolism pathways, which were accompanied by enhanced clonogenic survival. This data was supported *in vivo*, in pre-treatment OAC tumour tissue. Tumour ATP5B expression, a marker of oxidative phosphorylation, was significantly increased in patients who subsequently had a poor pathological response to neoadjuvant CRT. This suggests for the first time, a role for specific mitochondrial alterations and metabolic remodelling in the radioresistance of OAC.

## Introduction

Cancer of the oesophagus occurs worldwide [1] and despite recent advances in diagnosis and treatment, prognosis is poor with 5-year survival rates between 20 and 40% [2]. Recently, there has been a dramatic epidemiological shift in the incidence of oesophageal adenocarcinoma (OAC), which is now the dominant subtype in many western populations [3], [4]. Neoadjuvant chemoradiation therapy (CRT) followed by surgery is increasingly the standard of care for oesophageal cancer patients treated with curative intent [5], [6]. There is significant evidence that the tumour response to neoadjuvant CRT is the best predictor of both disease-free and overall survival, with 5-year survival rates increased to 60% for patients achieving a complete pathological response (pCR) [7], [8].

Radiation therapy exerts its effects locoregionally and is important for local tumour control. Whilst higher pCR rates are achieved with CRT than with induction chemotherapy alone [9], [10], only ∼30% of patients achieve a pCR [7], [11]. The elucidation of underlying molecular mechanisms of radioresistance and the identification of biomarkers that predict those patients who will respond to treatment is imperative to improving both the efficacy of treatment and survival rates.

DNA is the primary target of ionising radiation (IR). The mitochondrial genome is the only other cellular source of genetic material and is extremely susceptible to oxidative damage [12], making it a likely target for IR-induced damage. In addition to their essential role in regulating ATP production via oxidative phosphorylation, mitochondria also regulate other key processes including cell death and the cellular redox environment [13], both of which have been implicated in the cellular response to radiation. IR induces a complex cellular response involving multiple pathways [14], [15], therefore, mechanisms of radioresistance are likely to be polygenic in nature and energy-requiring. Modification of energy metabolism is an emerging hallmark of cancer [16]. Whilst the best known alteration is the so called ‘Warburg effect’, whereby tumour cells demonstrate a reliance on glycolysis even in the presence of O_2_ [17], there is accumulating evidence to suggest that a wide spectrum of bioenergetic profiles exist in human cancers [18], [19]. This suggests that the ability of a tumour to adapt and remodel energy metabolism in response to both internal and external stimuli, may be an important mechanism underlying cancer initiation, progression and response to anti-cancer therapy. The central role of the mitochondria in regulating energy production suggests that alterations in mitochondrial function and energy production may be important for the cellular response to radiation, however, this remains under-investigated.

In this study, we demonstrate alterations in mitochondrial function and bioenergetics in both an isogenic cell line model of radioresistant OAC and OAC tumour biopsies, supporting a role for mitochondrial dysfunction in the radioresistance of OAC.

## Materials and Methods

### Cells lines and cell culture

The human oesophageal adenocarcinoma cell line OE33 was obtained from the European collection of cell cultures. OE33 P and OE33 R cells were generated, cultured and characterised in our laboratory as previously described [20].

### Irradiation

Irradiation was performed using a Gulmay Medical X-ray generator, (RS225) (Gulmay Medical), at a dose rate of 3.25 Gray (Gy) per min.

### ROS and mitochondrial mass measurement

ROS and mitochondrial mass were measured by high content screening using CM-H_2_DCFDA (20 µM) and MitoTracker Green FM (300 nM) fluorescent probes (Invitrogen), respectively. Cells were seeded in 96-well plates at a density of 15,000 cells/well. Medium was removed and cells were incubated for 30 min at 37°C with respective probes in staining buffer (130 mM NaCl, 5 mM KCl, 1 mM Na_2_HPO_4_, 1 mM CaCl_2_, 1 mM MgCl_2_ and 25 mM Hepes, pH 7.4). Cells were counterstained with Hoechst 33342 (Invitrogen) nuclear stain (3 µg/mL in staining buffer) for 30 min at 37°C. Cells were analyzed on an In Cell analyzer 1000 (GE Healthcare). Five fields of view/well were acquired using a 10× objective. Nuclear staining with Hoescht 33342 was detected using an excitation filter of 360 nm and emission filter of 535 nm. CM-H_2_DCFDA and Mitotracker green FM were detected using an excitation filter of 480 nm and emission filter of 535 nm. Mean cellular fluorescence intensity was used to calculate ROS and mitochondrial mass using In Cell analyzer 1000 workstation image analysis software, which allowed normalization of the data to cell numbers.

### Random Mutation Capture (RMC) assay

The RMC assay was carried out on cells at a confluency of 80% in T25 cm^2^ flasks. DNA isolation, *Taq* ^α^I digestion, qPCR and sequencing was carried out as previously described [21]. All mutations identified occur at a single 5′-TCGA-3′ sequence, located at base pairs 1216 to 1220 of the mitochondrial genome.

### RNA isolation

Total RNA was isolated using RNeasy Plus Mini Kit (Qiagen) as per the manufacturer’s instructions. RNA was quantified using a Nanodrop 1000 spectrophotometer v3.3 (Thermo Scientific).

### Mitochondria and metabolism PCR gene arrays

RNA was reversed transcribed to cDNA using a First Strand cDNA synthesis kit (Qiagen), as per the manufacturer’s instructions. cDNA samples were applied to RT^2^ Profiler PCR Arrays (Qiagen), and qPCR was performed as per the manufacturer’s instructions using an ABI Prism 7900 HT real-time thermal cycler (Applied Biosystems). Data was analyzed by the 2^−ΔΔCt^ method using SDS RQ 1.2 relative quantification software (Applied Biosystems). One sample was set as the calibrator for the analysis.

### Transmission electron microscopy

OE33 P and OE33 R cells (pre and 24 h post irradiation with 2 Gy) were fixed with gluteraldehyde (3% in 0.05 M Potassium Phosphate buffer, pH 6.8) for 1 h at room temperature. Samples were processed and analyzed using a Jeol JEM2100 LaB6 (operated at 100 Kv). Digital images were obtained using an AMT XR80 capture system and ImageJ software.

### Crystal violet assay

Cells were fixed with 1% gluteraldehyde (Sigma-Aldrich) for 20 min at room temperature. The fixative was removed and cells were stained with crystal violet (0.1% in PBS) for 30 min at room temperature. Cells were washed with H_2_O and allowed to air dry. Cells were incubated with Triton X (1% in PBS) on a shaker for 15 min at room temperature. Absorbance was read at 595 nm using a Wallac Victor2 1420 multi-label counter (Perkin Elmer).

### Intracellular ATP measurement

OE33 P and OE33 R cells (pre and 24 h post irradiation with 2 Gy) were seeded in 96-well white-walled plates (15,000 cells/well) and allowed to adhere overnight. Relative intracellular ATP levels were measured using the luminescence-based ATPLite assay system (Perkin Elmer), as per the manufacturer’s instructions. Luminescence was measured using a Wallac Victor2 1420 multilabel counter. An additional plate was set up concurrently and a crystal violet assay was performed to normalize ATP measurements to cell number.

### OCR and ECAR measurements

Oxygen consumption rates (OCR) and extracellular acidification (ECAR) rates were measured before and after treatment with oligomycin (2 µg/mL, Seahorse Biosciences), antimycin (2.6 µM, Seahorse Biosciences) and 2-Deoxyglucose (55 µg/mL, Sigma), using a Seahorse XF24 analyzer (Seahorse Biosciences). Briefly, irradiated and ‘mock’ irradiated OE33 P and OE33 R cells were seeded at 18,000 and 20,000 cells/well, respectively, in a 24-well cell culture XF microplate (Seahorse Biosciences) and allowed to adhere overnight. Cells were washed with assay medium (unbuffered DMEM supplemented with 5 mM glucose, pH 7.4) before incubation with assay medium (0.5 mL) for 1 h at 37°C in a CO_2_- free incubator. Four baseline OCR and ECAR measurements were taken over 28 min. Two OCR and ECAR measurements were taken over 14 min following injection of antimycin, oligomycin or 2-deoxyglucose. All measurements were normalized to cell number using the crystal violet assay. ATP turnover was calculated by dividing the percentage of OCR linked to mitochondrial respiration by the percentage of OCR linked to ATP synthesis. Proton leak was calculated by subtracting the % of OCR linked to ATP turnover from 100%.

### Clonogenic assay

Cells were collected by trypsinisation, counted and seeded at optimised cell seeding densities (1.5×10^3^–3.0×10^4 ^cells/well) in 6-well plates and allowed to adhere overnight. Cells were treated with oligomycin (2 µg/mL) or DMSO in complete medium for 1.5 h. Media was removed and replaced with complete medium, and cells were incubated at 37°C in 5% CO_2_/95% air for 9–12 days. Colonies were fixed and counted as described previously [20].

### Ethics statement, patients, sample collection and treatment

Following ethical approval (Joint St James’s Hospital/AMNCH ethical review board) and written informed consent, diagnostic biopsy specimens were taken from patients with a diagnosis of operable OAC, by a qualified endoscopist prior to neoadjuvant therapy. Histologic confirmation of tumour tissue in biopsies was performed by a pathologist using routine hematoxylin and eosin staining. Patients received a complete course of neoadjuvant CRT. Chemotherapy consisted of 2 courses of 5- fluorouracil (5-FU) and cisplatin, as previously described [22]. Patients received 40.05 Gy in 15 daily fractions (2.67 Gy/fraction) over 3 weeks as previously described [22]. Surgical resection was performed within 1 month of completing the CRT regimen. All resected esophagectomy specimens were assessed by an experienced pathologist. Tumour response to treatment was assigned 1 of 5 tumour regression grades (TRG), as previously described [23]. Good responders were classified as patients achieving a TRG of 1 or 2, whilst poor responders were classified as patients having a TRG of 3, 4 or 5, as previously described [24].

### Immunohistochemistry

Tissue microarrays (TMA) were constructed from formalin-fixed paraffin-embedded pre-treatment tumour biopsies using 0.6 mm cores. Immunohistochemistry was performed using a Vectastain ABC Kit (Elite), as per the manufacturer’s instructions. Slides were deparaffinised, rehydrated and heated-antigen retrieval was performed using Trilogy solution (Cell Marque Corporation). Endogenous peroxidase activity was blocked using hydrogen peroxide (3%) for 30 min and sections were blocked using horse/goat serum and incubated with either mouse-anti-human ATP5B antibody (Santa Cruz Biotechnology, 1∶1000 dilution), mouse-anti-human HSP60 (Abcam, 1∶400 dilution), rabbit-anti-human PKM2 (Abgent, 1∶100 dilution) or rabbit-anti-human GAPDH (AbDserotec, 1∶100 dilution) for 1 h at room temperature. Sections were then incubated with secondary antibody/horseradish peroxidise for 30 min at room temperature. Diaminobenzidine (Sigma) was used to visualize staining and sections were counter-stained with haematoxylin, dehydrated and mounted. Stained sections were scanned using a scanscope XT digital scanner and ImageScope software (Aperio Technologies). Expression was assessed by scoring positive cell count and intensity in the epithelium and stroma by 2 reviewers who were blinded to the TRG status of the patient. Positive cell count was assessed using 6 categories (0%, 10%, 25%, 50%, 75%, 90% and 100%). Intensity was assessed using a scale of 0, 1, 2 and 3 which correlated with negative, weak, medium and strong staining, respectively.

### ATP5B qPCR

Total RNA (1 µg) was reverse transcribed to cDNA using random hexamers (Invitrogen) and BioScript (Bioline). qPCR was performed using a TaqMan primer probes (Applied Biosystems) and 18S was used as an endogenous control for data normalization. Data was analysed using SDS 2.3 and SDS RQ 1.2 relative quantification software (Applied Biosystems). One control sample was set as the calibrator for the analysis.

### Western blotting

Cells were lysed in RIPA buffer (50 mM Tris, pH 7.5, 150 mM NaCl, 2 mM EDTA, pH 8.0, 0.5% Triton X-100, protease inhibitor cocktail, 1 mM PMSF and 1 µM Na_3_VO_4_). Sample protein concentrations were quantified using the BCA assay (Pierce). Proteins (30 µg) were resolved on a 12% polyacrylamide gel and transferred to polyvinylidene fluoride membranes. Immunoblots were incubated with mouse anti-human ATP5B antibody (Santa Cruz Biotechnology), 1∶500 dilution or rabbit anti-human β-actin (Abcam), 1∶2,000 dilution, followed by incubation with HRP-labelled donkey anti-mouse (R&D systems) or goat anti-rabbit (Santa Cruz Biotechnology) IgG antibodies, respectively. Detection was performed using SuperSignal West Pico chemiluminescent substrate kit (Thermo Scientific). Densitometric analysis was performed using ImageJ software. β-actin was used as an endogenous control for data normalization.

### Statistical analysis

Statistical analysis was carried out using GraphPad InStat v3 (GraphPad software Inc). Significance was determined by two-tailed Students *t*-test for normally distributed data. For all statistical analysis, differences were considered to be statistically significant at *p*<0.05.

## Results

### OE33 R cells demonstrate increased mutagenesis and altered mitochondrial function

To investigate the role of mitochondrial alterations in the radioresistance of OAC, we used an isogenic cell line model of radioresistant OAC, previously generated in our laboratory [20]. The radioresistant OE33 R subline, which was generated by chronic irradiation with clinically-relevant fractionated doses of 2 Gy X-ray radiation, displays enhanced resistance to radiation when compared with its radiosensitive OE33 parent (OE33 P) cell line [20]. These two cell lines, of the same origin but with distinctly different radiosensitivities, provide a unique model with which to investigate the molecular determinants of response to radiation in OAC.

**Figure 1:**
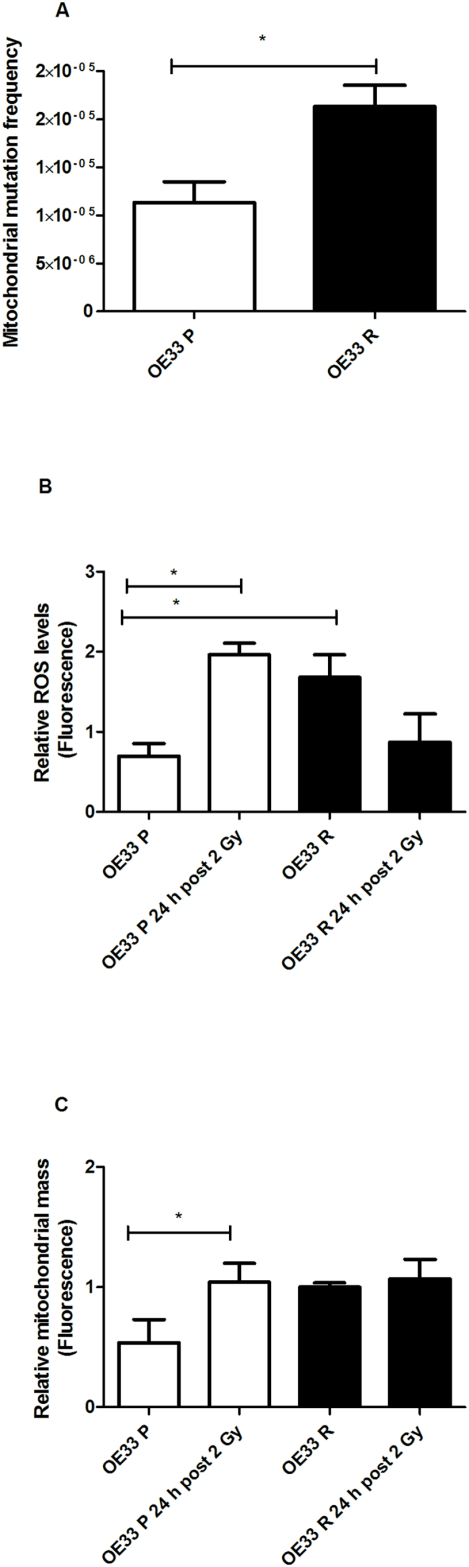
Radioresistant OE33 R cells have increased mitochondrial mutagenesis and altered mitochondrial function. (**A**) OE33 R cells demonstrate significantly elevated basal levels of random mitochondrial mutations, when compared to OE33 P. (**B**) OE33 R cells have significantly increased basal ROS levels, when compared to OE33 P. ROS levels are significantly increased in OE33 P cells at 24 h post irradiation with 2 Gy. (**C**) Mitochondrial mass is significantly increased in OE33 P cells at 24 h post irradiation with 2 Gy, this effect is not seen in OE33 R. Data are presented as mean±SEM from 3 independent experiments. Statistical analysis was performed by 2-tailed Student’s *t*-test, *P<0.05.

Given the close proximity of the mitochondria to potential endogenous sources of ROS via the electron transport chain, we assessed the basal frequency of random mutations in the mitochondrial genome of both radiosensitive OE33 P and radioresistant OE33 R cells, using the random mutation capture assay. OE33 R cells demonstrated a significant (*p* = 0.033) increase in basal levels of random mitochondrial mutations, when compared to OE33 P cells (Figure 1A). All mutations occurred within a known 4-bp sequence located within the 12S ribosomal gene. Sequencing data showed a predominance of C>T and G>T transitions in OE33 P cells, with A>G transitions also present in OE33 R cells. There was no significant alteration in random mutation levels in either cell line at 24 h post irradiation (data not shown).

We then investigated if this increased basal mitochondrial mutagenesis in OE33 R was accompanied by alterations in mitochondrial function. ROS and mitochondrial mass, which are surrogates of mitochondrial function were assessed both basally and at 24 h post irradiation with 2 Gy. This time point was chosen as we have previously demonstrated alterations in pathways implicated in the radioresponse at this time point [20]. Basal ROS levels were significantly increased in OE33 R, when compared to OE33 P (*p* = 0.048) (Figure 1B). Interestingly, at 24 h post irradiation ROS were significantly elevated in OE33 P cells (*p* = 0.035), an effect not observed in OE33 R cells. Increases in ROS in OE33 P following irradiation was coupled with a significant increase in mitochondrial mass (*p* = 0.043) (Figure 1C), which was not observed in OE33 R cells. These findings suggest that mitochondrial function is altered in OE33 R cells.

### Mitochondrial number and morphology is altered in OE33 R cells

**Figure 2:**
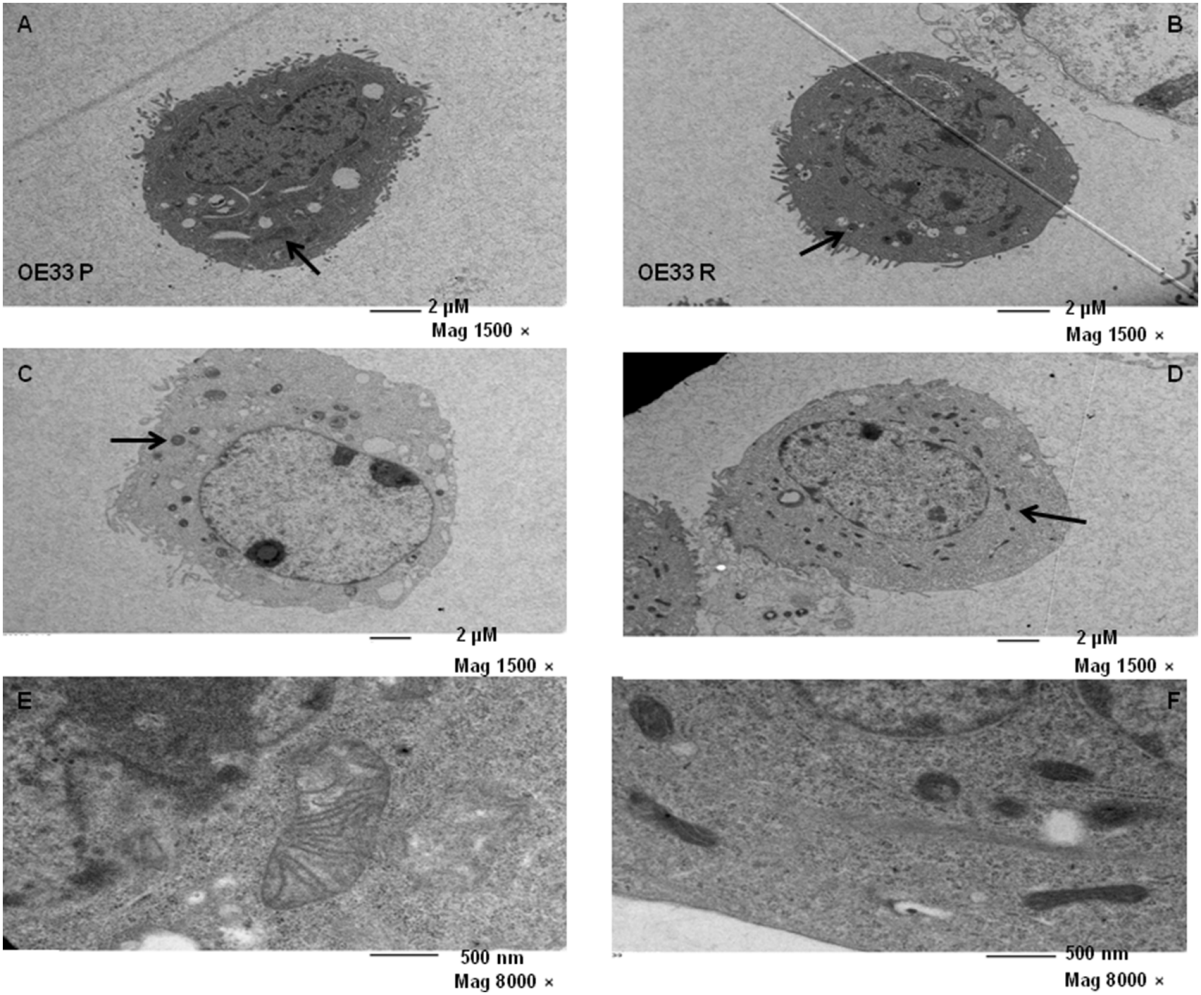
OE33 R cells have increased basal numbers of mitochondria and display altered morphology. The ultrastructure of OE33 P (A, C and E) and OE33 R cells (B, D and F) was assessed basally (A, B, E, F) and at 24 h post 2 Gy (C and D) by TEM. Representative images are shown. Black arrows point to mitochondria.

The ultrastructure of OE33 P and OE33 R cells was assessed by transmission electron microscopy, basally and at 24 h post irradiation with 2 Gy. OE33 R cells appeared to have increased basal numbers of mitochondria, when compared to OE33 P cells (Figures 2A, B). The number of mitochondria in OE33 P appeared to increase at 24 h post irradiation, when compared to basal levels (Figure 2C), supporting the previously demonstrated alterations in mitochondrial mass. Furthermore, the mitochondrial morphology in irradiated OE33 P cells was altered with a similar phenotype to that seen in OE33 R cells. Radiation did not appear to alter mitochondrial number or morphology in OE33 R cells (Figure 2D).

At high power magnification, the basal mitochondrial morphology in OE33 P and OE33 R demonstrated striking alterations. Mitochondria in OE33 P cells displayed the traditional mitochondrial structure, with cristae clearly visible (Figure 2E). In contrast, OE33 R mitochondria were much more elongated and condensed (Figure 2F). This increased density suggests that the mitochondria in OE33 R cells may have increased biochemical and metabolic activity. These alterations further support a role for mitochondrial dysfunction in the radioresistance of this model.

### OE33 R cells have increased energy and mitochondrial metabolism rates

**Figure 3:**
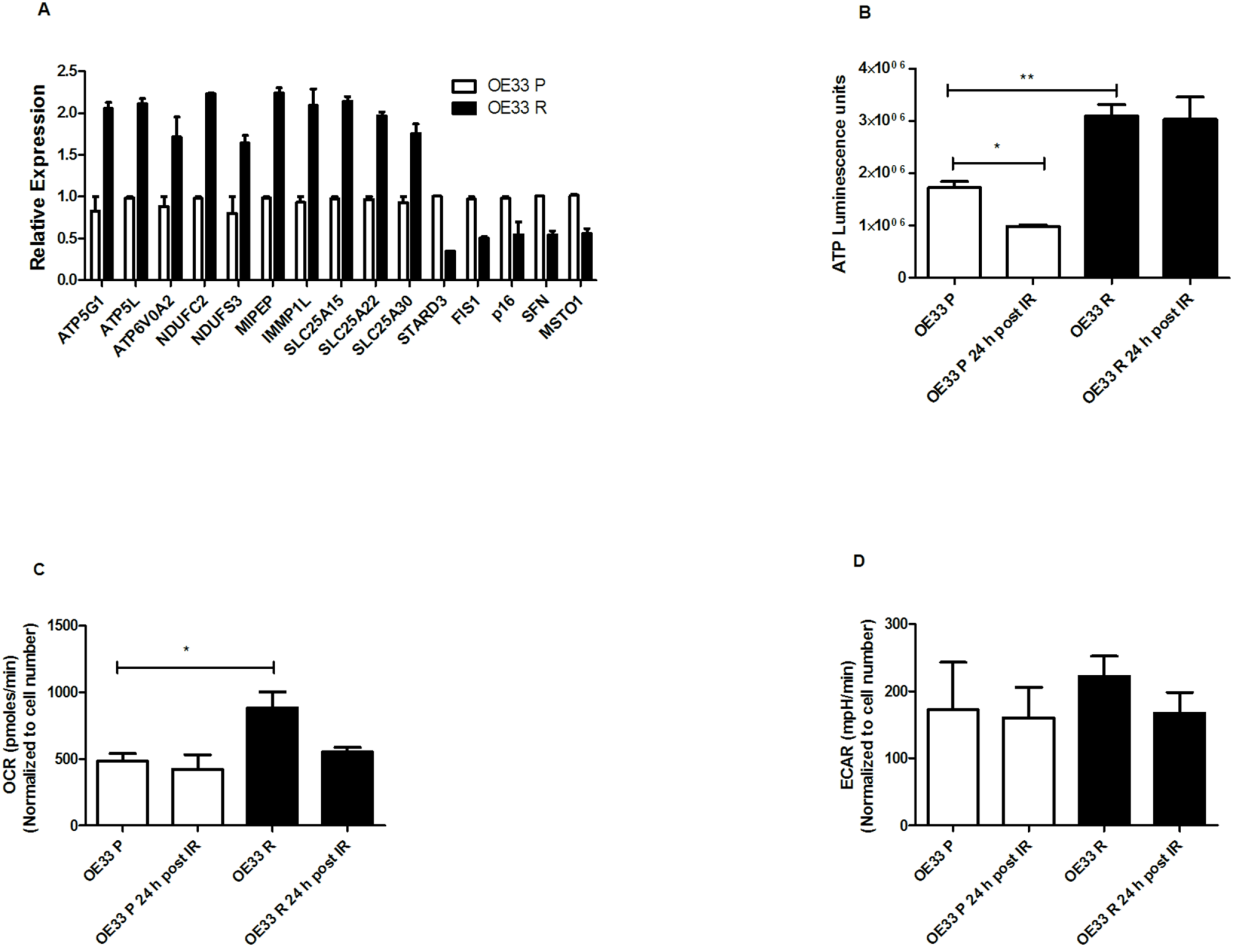
OE33 R cells display alterations in mitochondrial-associated gene expression, total cellular energy and energy metabolism pathways. (A) Basal expression of 15 mitochondrial function and energy metabolism-associated genes were altered 1.5-fold in OE33 R cells, when compared to OE33 P. Data are presented as mean ± SEM from 2 independent experiments. (B) OE33 R cells have significantly increased basal intracellular ATP levels, when compared to OE33 P. ATP levels significantly decrease in OE33 P cells at 24 h post irradiation with 2 Gy, when compared to basal levels. Data are presented as mean ± SEM from 1 independent experiment. (C) Basal oxygen consumption rates (OCR) are significantly increased in OE33 R cells, when compared to OE33 P. Data are presented as mean ± SEM from 4 independent experiments. (D) OE33 R and OE33 P cells demonstrate similar extracellular acidification rates (ECAR). Data are presented as mean ± SEM from 4 independent experiments. Statistical analysis was performed by 2-tailed Student’s *t*-test, *P<0.05, **P<0.01.

The observed alterations in mitochondrial function and ultrastructural morphology in OE33 R cells were further supported at the gene level, with basal expression of 15 genes involved in regulating mitochondrial function and energy metabolism altered ≥1.5-fold in OE33 R cells, when compared to OE33 P (Figure 3A). Interestingly, all 5 altered energy metabolism-associated genes (namely, *ATP5G1*, *ATP5G3*, *ATPV0A2*, *NDUFC2* and *NDUFS3*) were increased in OE33 R cells, supporting increased metabolic activity in these cells. To investigate if this correlated with increased energy, we measured intracellular ATP levels in both cell lines, basally and at 24 h post irradiation with 2 Gy. Supporting what we had seen at the ultrastructural and gene expression level, OE33 R cells had significantly increased basal intracellular ATP levels (*p* = 0.004), when compared to OE33 P cells (Figure 3B). Irradiation with 2 Gy significantly reduced ATP levels at 24 post irradiation in OE33 P cells (*p* = 0.004), however, this was not observed in OE33 R cells. This suggests that OE33 R cells can more efficiently maintain intracellular energy levels following cytotoxic insult.

To investigate if the increased ATP levels in OE33 R were due to alterations in energy metabolism, we measured the two major energy pathways, oxidative phosphorylation and glycolysis, in OE33 P and OE33 R using the Seahorse XF analyzer. This allows the simultaneous measurement of OCR, which is a measure of oxidative phosphorylation and the ECAR, a product of glycolysis, in live cells in real-time. OE33 R cells had a significantly increased basal OCR when compared to OE33 P cells (*p* = 0.02) (Figure 3C), suggesting a mechanism for the increased levels of intracellular ATP in radioresistant cells. OE33 R cells demonstrated a trend towards increased basal ECAR, when compared to OE33 P (222 versus 173 mpH/min, respectively), however, this was not statistically significant (Figure 3D). Taken together, these data suggest that OE33 R cells are more metabolically active, which may be a mechanism underlying their radioresistance.

### OE33 R cells have altered bioenergetics

**Figure 4:**
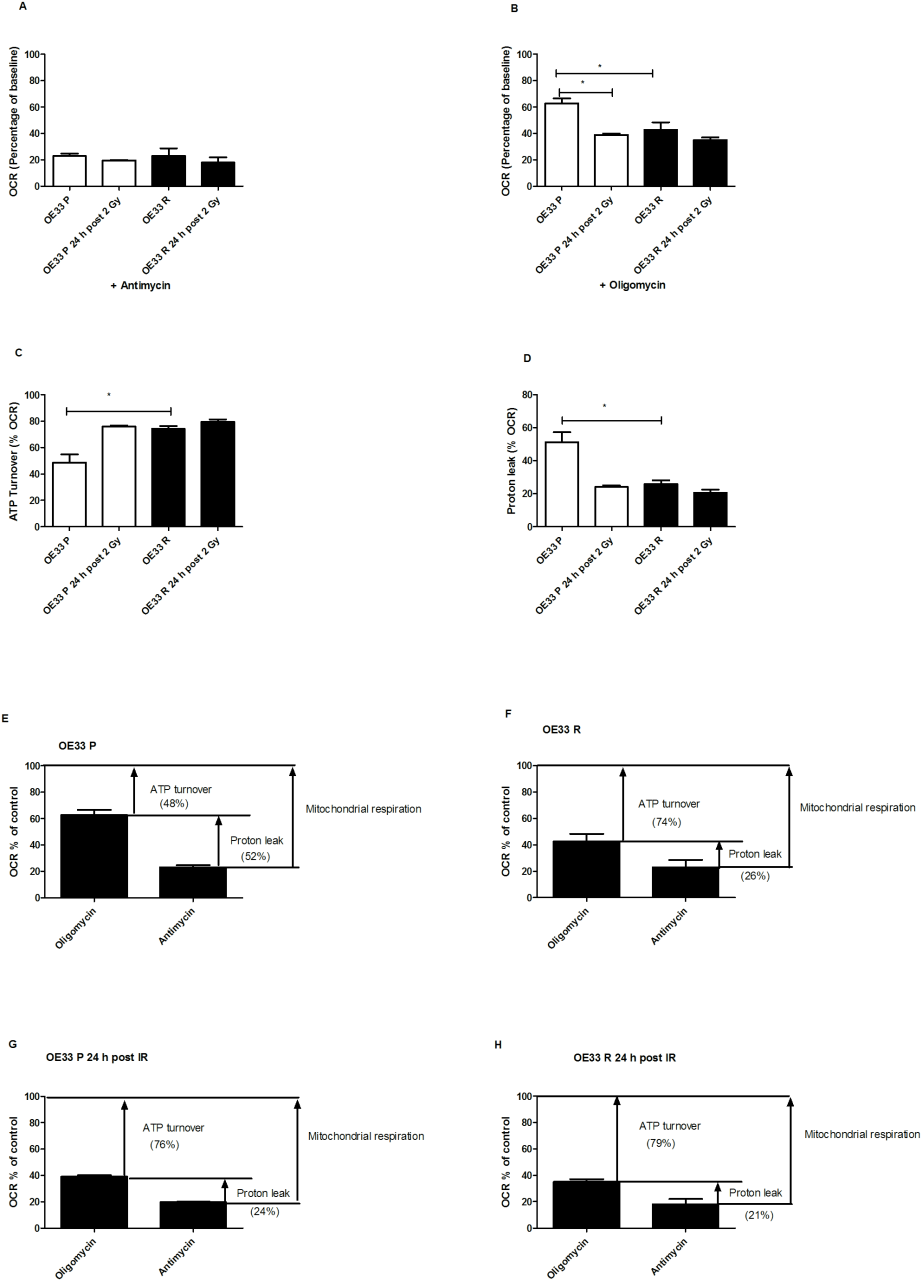
OE33 R cells have higher ATP turnover and reduced proton leak. (**A**) OE33 P and OE33 R cells demonstrate similar sensitivity to antimycin (2.6 µM). (**B**) OE33 R cells were significantly more sensitive to the effects of oligomycin (2 µg/mL) on OCR, when compared to OE33 P cells. At 24 h post 2 Gy, OE33 P cells were significantly more sensitive to the effects of oligomycin on OCR, when compared to unirradiated cells. (C) OE33 R cells demonstrate significantly higher percentage of mitochondrial respiration coupled to ATP synthesis, when compared to OE33 P. (**D**) OE33 R cells demonstrate significantly lower proton leak, when compared to OE33 P. Data are presented as mean± SEM from at least 3 independent experiments. Statistical analysis was performed by 2-tailed Student’s *t*-test, *P<0.05. The contribution of mitochondrial respiration to ATP synthesis in OE33 P (**E**), OE33 R (**F**) at basal level and at 24 h post irradiation with 2 Gy (**G** and **H**).

To further investigate the alterations in oxidative phosphorylation in OE33 R cells, we treated OE33 P and OE33 R cells with two specific inhibitors of the electron transport chain, antimycin and oligomycin. Antimycin inhibits complex III, effectively inhibiting all mitochondrial respiration and therefore allows measurement of the proportion of cellular oxygen consumption that is related to mitochondrial respiration. Antimycin reduced OCR to similar levels in both unirradiated and irradiated OE33 P and OE33 R cells (Figure 4A), suggesting that mitochondrial respiration rates are similar in both cell lines. Antimycin-resistant rates reflect non-mitochondrial respiration, which includes cell surface oxygen consumption [25]. Cells were also treated with oligomycin, which inhibits complex V where the electron transport chain is coupled to ATP synthesis. Interestingly, OE33 R cells were significantly more sensitive to oligomycin, when compared to OE33 P cells, with oligomycin reducing OCR to ∼43% and ∼63% of baseline rates, respectively (Figure 4B). Irradiated OE33 P cells were also more sensitive to oligomycin, with OCR reduced a further 24% when compared to unirradiated cells (from 63% to 39%). This effect was not observed in OE33 R cells.

OE33 R cells had a significantly increased percentage of mitochondrial respiration linked to ATP turnover, when compared to OE33 P cells (*p* = 0.02) (Figure 4C), which supports the demonstrated alterations in intracellular ATP levels. OE33 R cells also had a significantly lower percentage of mitochondrial respiration accounted for by proton leak (*p* = 0.02), when compared to OE33 P cells (Figure 4D). Proton leak is an extremely energy-inefficient process, supporting the enhanced metabolic activity of OE33 R cells. Interestingly, irradiated OE33 P cells had a trend towards increased ATP turnover and decreased proton leak (*p* = 0.053), when compared to unirradiated controls, suggesting IR-induced metabolic changes in these cells. Combining these results (Figures 4E, F, G, H), the demonstrated alterations in ATP turnover and proton leak in OE33 R cells supports a role for altered bioenergetics in the radioresistance of this model.

**Figure 5:**
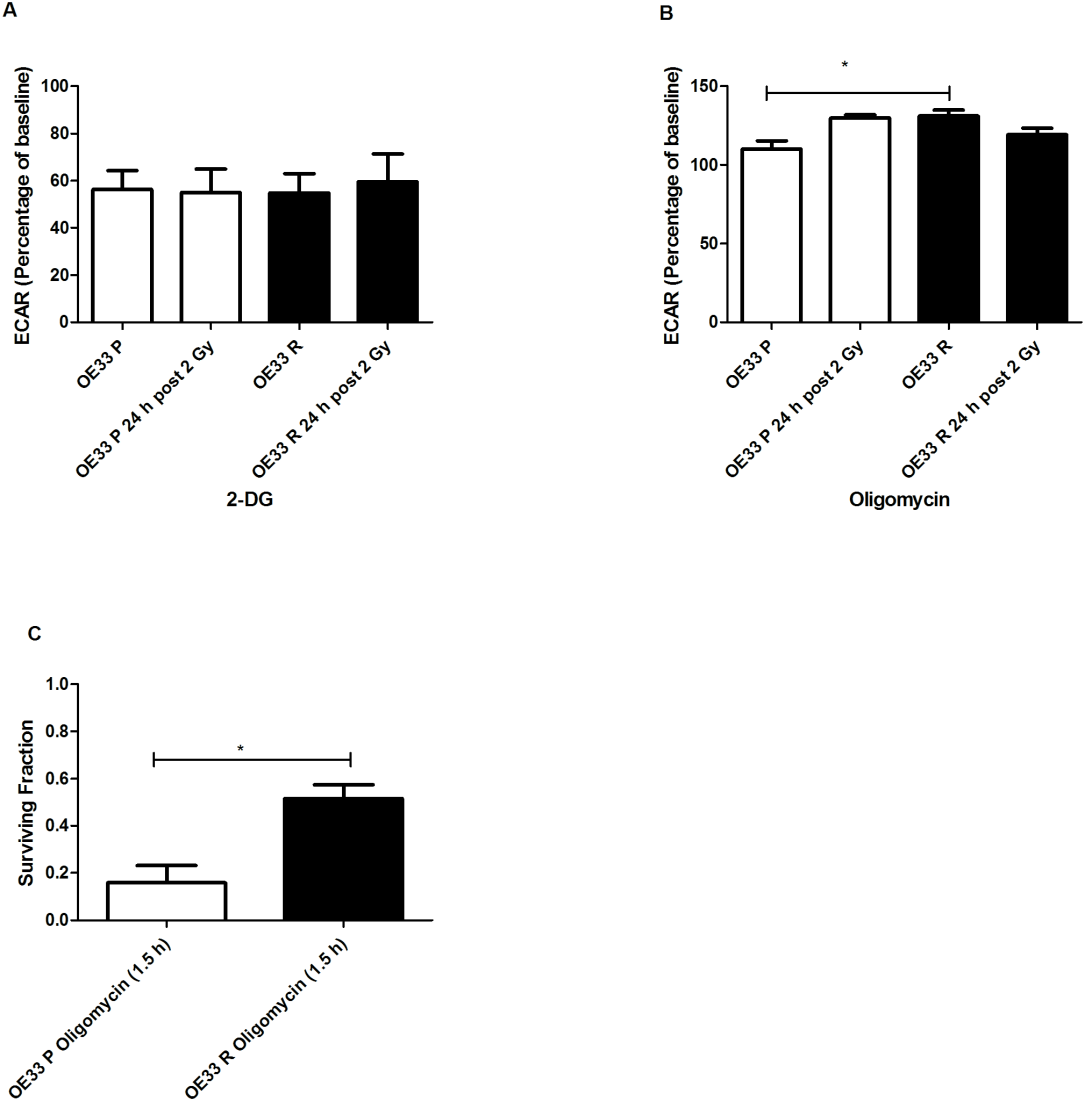
OE33 R cells are more metabolically robust. (**A**) OE33 P and OE33 R cells display similar sensitivities to the glycolytic inhibitor 2-deoxyglucose (55 µg/mL). (**B**) ECAR is significantly increased in OE33 R cells following oligomycin treatment (2 µg/mL), when compared to OE33 P. (**C**) OE33 R cells have significantly enhanced clonogenic survival following treatment with oligomycin (2 µg/mL) for 1.5 h, when compared to OE33 P cells. Data are presented as mean ± SEM from at least 3 independent experiments. Statistical analysis was performed by 2-tailed Student’s *t*-test, *P<0.05.

To investigate if alterations in glycolysis were involved in the radioresistance of OE33 R, we treated both OE33 R and OE33 P cells with the glycolytic inhibitor 2-deoxyglucose (2-DG) (Figure 5A). Treatment with 2-DG reduced ECAR rates to 56% and 55% from baseline, in OE33 P and OE33 R, respectively. A similar reduction in ECAR following 2-DG treatment was also seen in OE33 P and OE33 R cells at 24 h post irradiation with 2 Gy, with ECAR rates at 55% and 59% from baseline, respectively. This suggests that the contribution of glycolysis to energy metabolism is similar in both cell lines.

The reduction in OCR following oligomycin treatment (Figure 4B) was accompanied with an increase in ECAR in both cell lines, suggesting a switch from oxidative phosphorylation to glycolysis (Figure 5B). Interestingly, ECAR was significantly higher in OE33 R cells following oligomycin treatment when compared to OE33 P cells (*p* = 0.03), suggesting that radioresistant cells can more efficiently switch to glycolysis following inhibition of oxidative phosphorylation. Supporting this, OE33 R cells demonstrated significantly enhanced clonogenic survival (*p* = 0.02) following treatment with oligomycin for 1.5 h, when compared to OE33 P cells (Figure 5C).

### Energy metabolism is altered in tumours from OAC patients who have a poor response to neoadjuvant CRT

Having demonstrated alterations in both mitochondrial function and energy metabolism in an *in vitro* model of radioresistant OAC, we then investigated if metabolic alterations would indicate response to neoadjuvant CRT in OAC patient tumours. We examined expression of four protein markers associated with mitochondrial function and energy metabolism, HSP60, a mitochondrial chaperone protein, ATP5B a marker of oxidative phosphorylation and two markers of glycolysis, GAPDH and PKM2. Expression of these proteins was assessed in 23 OAC tumour tissue biopsies taken prior to neoadjuvant CRT, using tissue microarrays and immunohistochemistry. In this patient cohort, 30% were classified as ‘good’ responders (TRG 1 (*n* = 2) and 2 (*n* = 5)), whilst 70% were classified as ‘poor’ responders (TRG 3 (*n* = 8), 4 (*n* = 5) and 5 (*n* = 3)). There was no significant difference in age between good and poor responders. Patient cohort characteristics are outlined in Table 1.

**Table 1:** Patient cohort characteristics

	**(** ***n*** ** = 23)**
Male	16
Female	7
Age (years)^a^	61 (44–75)
**Clinical TNM Stage**	2 Patients NS
0	1
I	0
IIa	11
IIb	2
III	7
IV	0
**TRG**	
1	2
2	5
3	8
4	5
5	3
**Nodal Status**	
N0	7
N1	16

Abbreviations:^ a^Values given are mean (range); TNM, Tumour-node-metastasis clinical staging classification; NS, not specified; TRG, Tumour regression grade; N0, indicates lymph node metastasis negative; N1, lymph node metastasis positive.

**Figure 6:**
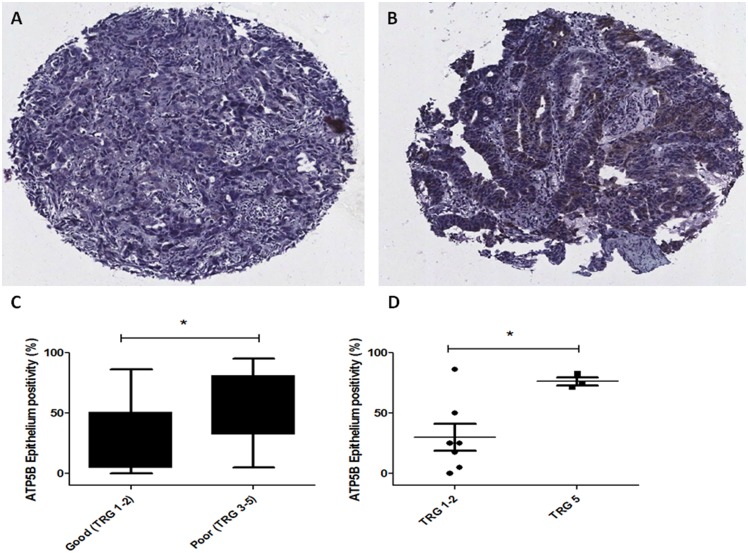
ATP5B expression is increased in the tumour epithelium of poor responders. ATP5B expression was assessed by immunohistochemistry in pre-treatment tumour biopsies from OAC patients who subsequently received neoadjuvant CRT (*n* = 23). Representative images of ATP5B staining in tumours from good (**A**) and poor (**B**) responders, magnification 10×. (**C**) ATP5B positivity in the epithelium is significantly increased in tumours of patients with a poor response to neoadjuvant CRT (TRG 3–5), when compared to good responders (TRG 1 and 2). (**D**) ATP5B positivity in the epithelium is significantly increased in tumours of patients with no evidence of regression (TRG 5), when compared to patients achieving a full (TRG 1) or major response (TRG 2). Statistical analysis was performed by unpaired 2-tailed Student’s *t*-test, *P<0.05.

Supporting the increased oxidative phosphorylation rates seen in radioresistant cells *in vitro*, expression of ATP5B was significantly higher (*p* = 0.043, 95% confidence interval −55.50 to −0.93) in the tumour epithelium of patients who had a poor response to neoadjuvant CRT (TRG 3–5), when compared to good responder patients (TRG 1–2) (Figure 6C). In addition, expression of ATP5B was significantly higher (*p*<0.032) in the tumour epithelium of patients with no evident regression following CRT (TRG 5), when compared to patients achieving a complete or major pathological response (TRG 1 and TRG 2, respectively) (Figure 6D). Similar stromal ATP5B expression was demonstrated in both good and poor responders (Figure S1). There was no significant association between epithelial ATP5B expression and lymph node status or overall survival (data not shown). Similarly to that demonstrated *in vitro*, expression of the glycolytic markers GAPDH and PKM2 was similar in both the epithelium and stroma of tumours from good and poor responders (Figure S1). This supports our *in vitro* data and suggests that alterations in mitochondrial function and energy metabolism, specifically enhanced oxidative phosphorylation, is associated with the tumour response to neoadjuvant CRT in OAC patients.

## Discussion

Whilst the overall prognosis of oesophageal cancer is poor, multi-modal approaches have improved outcomes in patients with locally advanced disease, with a 3-year survival of 58% reported for patients receiving neoadjuvant CRT in a recent multi-centre clinical trial [6]. The elucidation of both underlying molecular mechanisms and biomarkers of resistance to therapy is central to improving the efficacy of treatment and survival of OAC patients. Whilst there is strong evidence to support a role for altered mitochondrial function and energy metabolism in tumourigenesis, little is known about their role in the response to anti-cancer therapy, specifically radiation.

The mitochondrial genome is highly susceptible to oxidative damage, with mutations in the mitochondrial genome implicated in both the initiation [26] and progression [27] of cancer. Here, quantification of random mitochondrial mutations in an isogenic model of radioresistant OAC revealed that radioresistant OE33 R cells have significantly elevated basal levels of random point mutations, when compared to OE33 P. Given that DNA is the primary target for IR-induced damage, this may be explained by the chronic irradiation of the OE33 R cell line during its generation and is consistent with a previous study demonstrating increased mitochondrial mutagenesis in the progeny of irradiated cells [21]. Random mutations are not clonally expanded and therefore may account for the genetic heterogeneity demonstrated in tumours, which may have implications in the tumour response to treatment [28]. These data suggest that the burden of mitochondrial mutations in OE33 R cells may be important for their resistance to IR. Accompanying the increased frequency of random mutations, OE33 R mitochondria demonstrated alterations in function, number, size and morphology. Using high content screening analysis, radioresistant OE33 R cells demonstrated elevated basal levels of ROS, which may be the effector of the increased mitochondrial mutagenesis. IR-induced oxidative alterations can persist following the initial exposure [29] and may be passed on to unirradiated progeny [30]. Thus, the increased ROS levels may be due to the chronic irradiation of this cell line during its generation. Whilst ROS are critical mediators of IR-induced damage [31], paradoxically, ROS also play important roles in the signalling of survival and proliferative pathways [32] and thus may be important for the resistance of OE33 R cells to radiation. Interestingly, whilst basal ROS levels were increased in OE33 R cells, the converse was demonstrated post irradiation, with ROS levels elevated in OE33 P cells only. This suggests that the chronic state of oxidative stress in OE33 R cells renders them more resistant to subsequent oxidative stress insults. Supporting this, our previous work demonstrated significantly decreased levels of IR-induced DNA damage in OE33 R cells at 24 h post irradiation compared with radiosensitive OE33 P [20]. Interestingly, we have previously demonstrated that OE33 R cells maintain levels of the ROS scavenging enzyme glutathione following irradiation [20], suggesting that the difference in ROS levels between the OE33 P and OE33 R cell lines post irradiation may be due to alterations in ROS scavenging.

OE33 R cells appeared to have increased basal numbers of mitochondria. These mitochondria were much smaller when compared to OE33 P mitochondria, perhaps explaining why there were no significant alterations in overall mitochondrial mass. This supports previous studies [33], [34] that have demonstrated IR-induced mitochondrial biogenesis, with one study correlating mitochondrial content with the proportion of cells in the G2/M phase of the cell cycle [35]. We have previously demonstrated that OE33 R cells have significantly increased basal numbers of cells in G2/M phase [20], which may suggest a mechanism for this increased number of mitochondria. Radiation appeared to increase the numbers of mitochondria in OE33 P cells, which may partly account for the increased IR-induced ROS levels in these cells. Morphologically, the mitochondria of OE33 P and OE33 R were starkly altered, with OE33 R mitochondria more elongated and condensed. This supports a previous study which demonstrated similar morphological alterations, with mitochondria elongated and condensed in an *in vitro* isogenic model of chemoresistant colon cancer [36], suggesting mitochondrial alterations as a common mechanism of resistance to cytotoxic agents.

The mitochondria are the powerhouse of the cell. Given the demonstrated alterations in mitochondrial function, number and morphology in OE33 R cells, it was not surprising that perturbations in energy levels and energy metabolism were also demonstrated. Radioresistant OE33 R cells had increased intracellular ATP levels both basally and following irradiation, suggesting increased energy demand in these cells. Supporting this, OE33 R cells were demonstrated to be more metabolically active, with significantly increased basal oxidative phosphorylation rates. This supports the functionality of the mitochondria in these cells and, again, may explain the increased basal ROS levels. Given the polygenic nature of radioresistance mechanisms, most of which are likely to require ATP, this suggests that the increased ATP demand is a driving force behind the metabolic alterations in these cells. This supports a previous study, which demonstrated that the maintenance of intracellular ATP levels following irradiation was associated with a radioresistant phenotype in head and neck cancer [37]. ATP has previously been demonstrated to reduce radiation-induced DNA damage and inflammatory cytokine production [38], which may suggest a mechanism for this radioprotective effect. Interestingly, increased ATP levels have also been implicated in the resistance of colon cancer cells to chemotherapy [36], perhaps suggesting a common mechanism of resistance.

In addition to being more metabolically active, the bioenergetics of OE33 R cells were also altered, with a significantly higher proportion of mitochondrial respiration contributed to ATP synthesis, supporting the elevated intracellular ATP levels in these cells. Interestingly, irradiated OE33 P cells showed a similar contribution of mitochondrial respiration to ATP synthesis, although this was not reflected at the intracellular ATP level. These data support a previous study that correlated increased oxidative phosphorylation and ATP levels with radioresistance in glioma cancer stem cells [39].

Whilst there was a trend towards increased basal glycolysis in OE33 R cells, this was not statistically significant. OE33 P and OE33 R cells displayed similar sensitivities to the glycolytic inhibitor 2-deoxyglucose, suggesting that both cell lines have a similar dependence on glycolysis. This is in contrast to a study by Zhou and colleagues [36] that demonstrated enhanced glycolytic rates as a mechanism for the chemoresistance of colorectal cells, although their demonstrated correlation between ATP levels and resistance to chemotherapy is in line with our data. Interestingly, the ECAR rate was significantly increased in OE33 R cells following inhibition of oxidative phosphorylation, which was coupled with enhanced clonogenic survival following treatment with oligomycin. This may suggest that radioresistant cells have a metabolic plasticity, which allows them to efficiently switch to alternative metabolic pathways if required.

It is acknowledged that this *in vitro* cell line model of radioresistant OAC is a model of acquired radioresistance. However, our *in vitro* data was supported *in vivo*, with the functional subunit of ATP Synthase, ATP5B, significantly increased in the tumours of OAC patients who were shown to have a poor response to CRT. We have previously demonstrated that OE33 R and OE33 P have similar sensitivities to cisplatin and 5-FU [20]. This suggests that the alterations in ATP5B expression *in vivo,* may be predominantly involved in the cellular response to radiation. Similarly to that demonstrated *in vitro*, expression of the glycolytic markers GAPDH and PKM2 were not significantly altered between good and poor responders. This supports the clinical applicability of this isogenic radioresistant model, and suggests that a metabolic shift to aerobic respiration may be a mechanism of resistance to neoadjuvant CRT in OAC tumours. Whilst oxidative phosphorylation was significantly increased in radioresistant cells *in vitro*, levels of ATP5B were similar in both OE33 P and OE33 R cells, basally and following irradiation with 2 Gy (Figure S2), suggesting that the increased mitochondrial respiration in OE33 R is likely to involve multiple proteins involved in this complex metabolic pathway.

The Warburg effect is considered a characteristic of cancer cells [17]. However, adding to the complexity of cancer metabolism, several studies have demonstrated active oxidative phosphorylation in numerous tumour types [18], [40], and there is even evidence of a metabolic symbiosis between hypoxic and oxygenated cells within the same tumour [19], supporting a spectrum of cancer bioenergetic profiles. This is supported in this study, where ATP5B, HSP60, GAPDH and PKM2 were all demonstrated to be significantly increased in the tumour epithelium, when compared to the stromal compartment in OAC tumour biopsies (Figure S3). Whilst this may be reflective of differences in proliferative capacity between the two tissue compartments, this data demonstrates that both oxidative phosphorylation and glycolysis are active in these tumours.

The ability of tumour cells to adapt and reprogram their metabolism in response to both internal and external factors is now recognised as a hallmark of cancer [41]. Aerobic respiration is the most efficient process of ATP generation, yielding 38 ATP molecules when compared to 2 ATP molecules generated per glucose molecule from glycolysis [42]. Given the potential for increased energy demand in radioresistant cells, this metabolic reprogramming may provide an advantage for surviving the cytotoxic effects of IR. In addition, this metabolic shift is likely to increase oxidative stress via the generation of free radicals from the electron transport chain. Increasing evidence suggests that a chronic state of oxidative stress facilitates tumour resistance to subsequent oxidative stress inducers via the development of a stable oxidative stress-resistant phenotype [43] and/or promotion of genomic instability [44]. Interestingly, evidence suggests that DNA repair pathways, such as Base Excision Repair (BER) and Mismatch Repair (MMR) may be induced in mitochondria during oxidative insult [45], [46]. We have previously demonstrated that levels of DNA repair genes, including genes of the BER and MMR pathways, are increased in the tumours of patients who have a poor response to CRT [24], supporting increased oxidative stress in tumours of poor responders. A role for mitochondrial dysfunction has been demonstrated in the persistence of oxidative stress and genomic instability [47], which may facilitate a metabolic plasticity that is important for tumour survival following radiation and chemotherapy.

This study demonstrates for the first time a role for mitochondrial dysfunction and metabolic reprogramming in the radioresistance of OAC. Elucidating the role of the mitochondria and energy metabolism in the response of tumours to radiation therapy may allow the prediction of response to treatment and the identification of novel strategies and drug combination to enhance the efficacy of treatment.

## Supporting Information

Figure S1 **Immunohistochemical staining in pre-treatment OAC tumours.** Stromal expression of (**A**) ATP5B, (**C**) HSP60, (**E**) GAPDH, (**G**) PKM2 and epithelial expression of (**B**) HSP60, (**D**) GAPDH, (**F**) PKM2 was assessed in pre-treatment OAC biopsies from patients who subsequently had a good (*n* = 7) or poor (*n* = 16) response to neoadjuvant CRT.
(TIF)

Figure S2 **ATP5B expression in OE33 P and OE33 R.** ATP5B expression was assessed in OE33 P and OE33 R, basally and at 6 h and 24 h post irradiation with 2 Gy by (**A**) qPCR and (**B**) western blotting. Data are presented as the mean ± SEM from 3 independent experiments. (**C**) Representative western blot.
(TIF)

Figure S3 **Metabolism is increased in the tumour epithelium in pre-treatment OAC tumours.** Expression (percentage positivity and intensity) of ATP5B (**A and B**), HSP60 (**C and D**), GAPDH (**E and F**) and PKM2 (**G and H**) was assessed in the stromal and epithelial compartments in pre-treatment OAC biopsies (*n* = 23). Statistical analysis was performed by paired 2-tailed Student’s *t*-test or Wilcoxon non-parametric test. **P<0.01, ***P<0.001.
(TIF)

File S1 (DOCX)
