# Supplementary figures and images for: Altered Mitochondrial Function and Energy Metabolism Is Associated with a Radioresistant Phenotype in Oesophageal Adenocarcinoma
Source: PLoS One. 2014 Jun 26;9(6):e100738. doi: 10.1371/journal.pone.0100738 (PMC4072695; doi:10.1371/journal.pone.0100738)

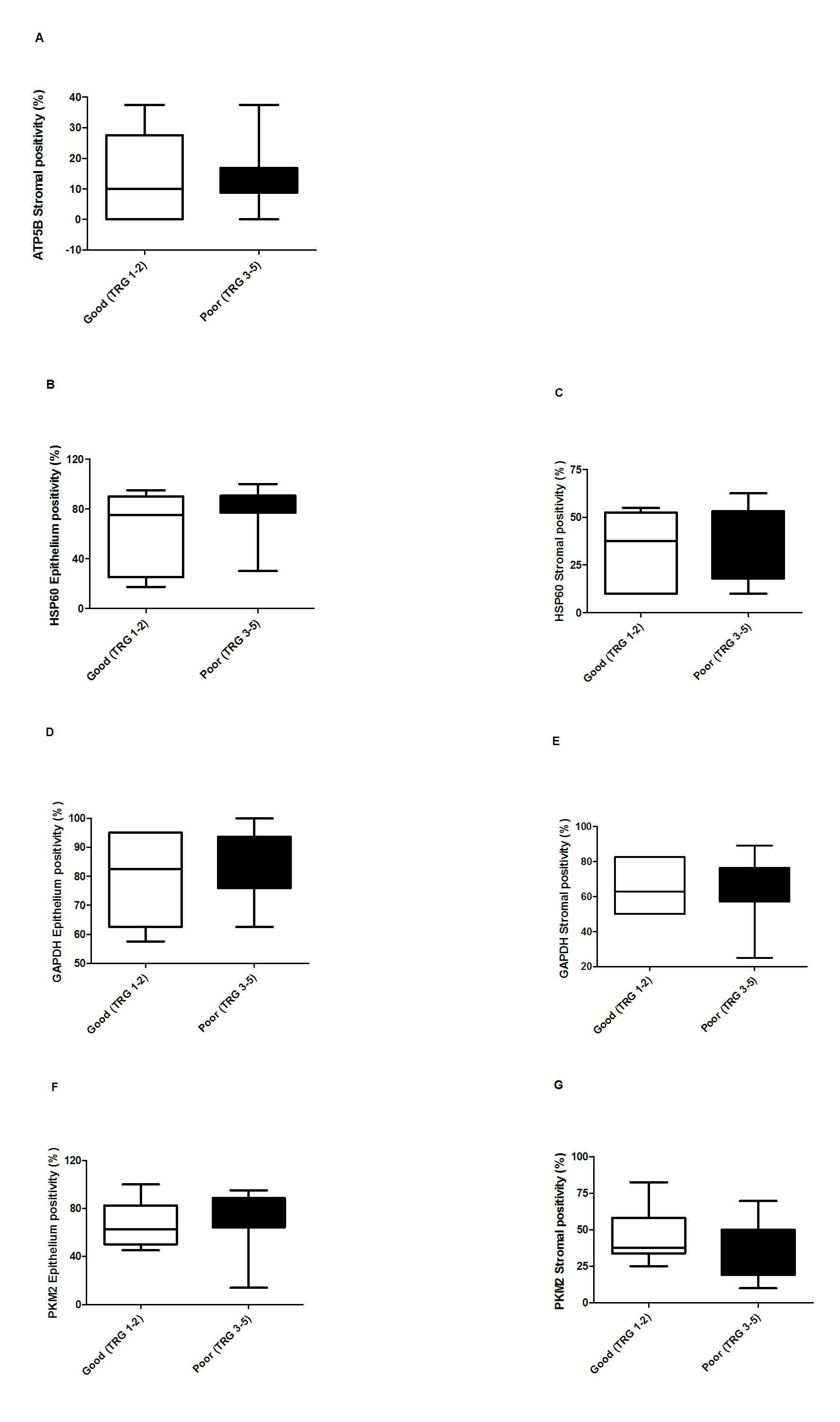

Supplement: Figure S1 — Immunohistochemical staining in pre-treatment OAC tumours. Stromal expression of (A) ATP5B, (C) HSP60, (E) GAPDH, (G) PKM2 and epithelial expression of (B) HSP60, (D) GAPDH, (F) PKM2 was assessed in pre-treatment OAC biopsies from patients who subsequently had a good (n = 7) or poor (n = 16) response to neoadjuvant CRT. (TIF) [file pone.0100738.s001.tif]

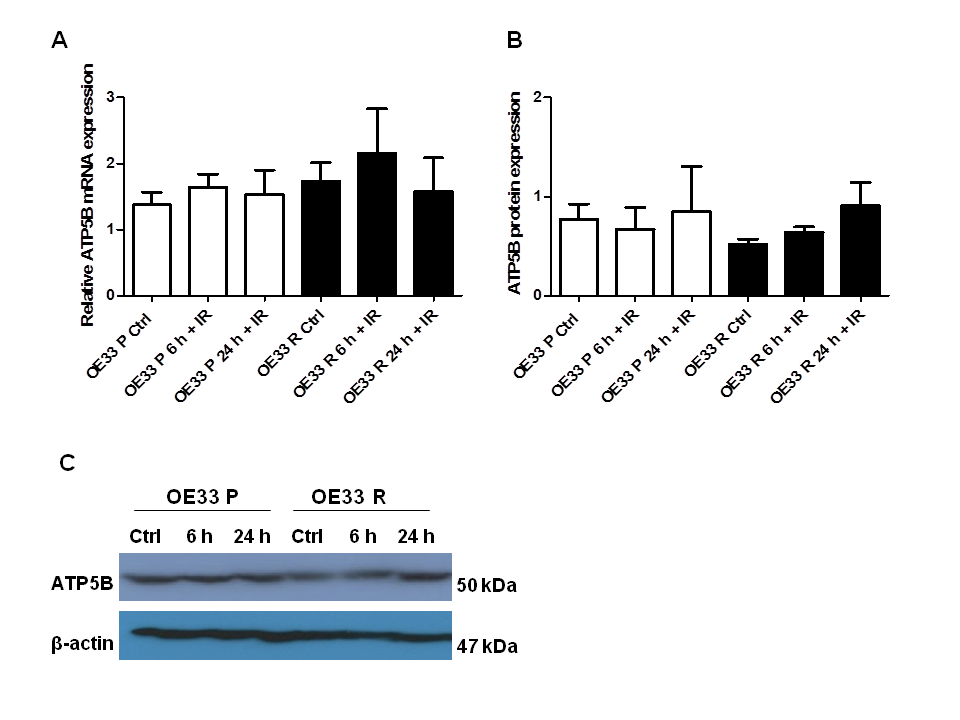

Supplement: Figure S2 — ATP5B expression in OE33 P and OE33 R. ATP5B expression was assessed in OE33 P and OE33 R, basally and at 6 h and 24 h post irradiation with 2 Gy by (A) qPCR and (B) western blotting. Data are presented as the mean ± SEM from 3 independent experiments. (C) Representative western blot. (TIF) [file pone.0100738.s002.tif]

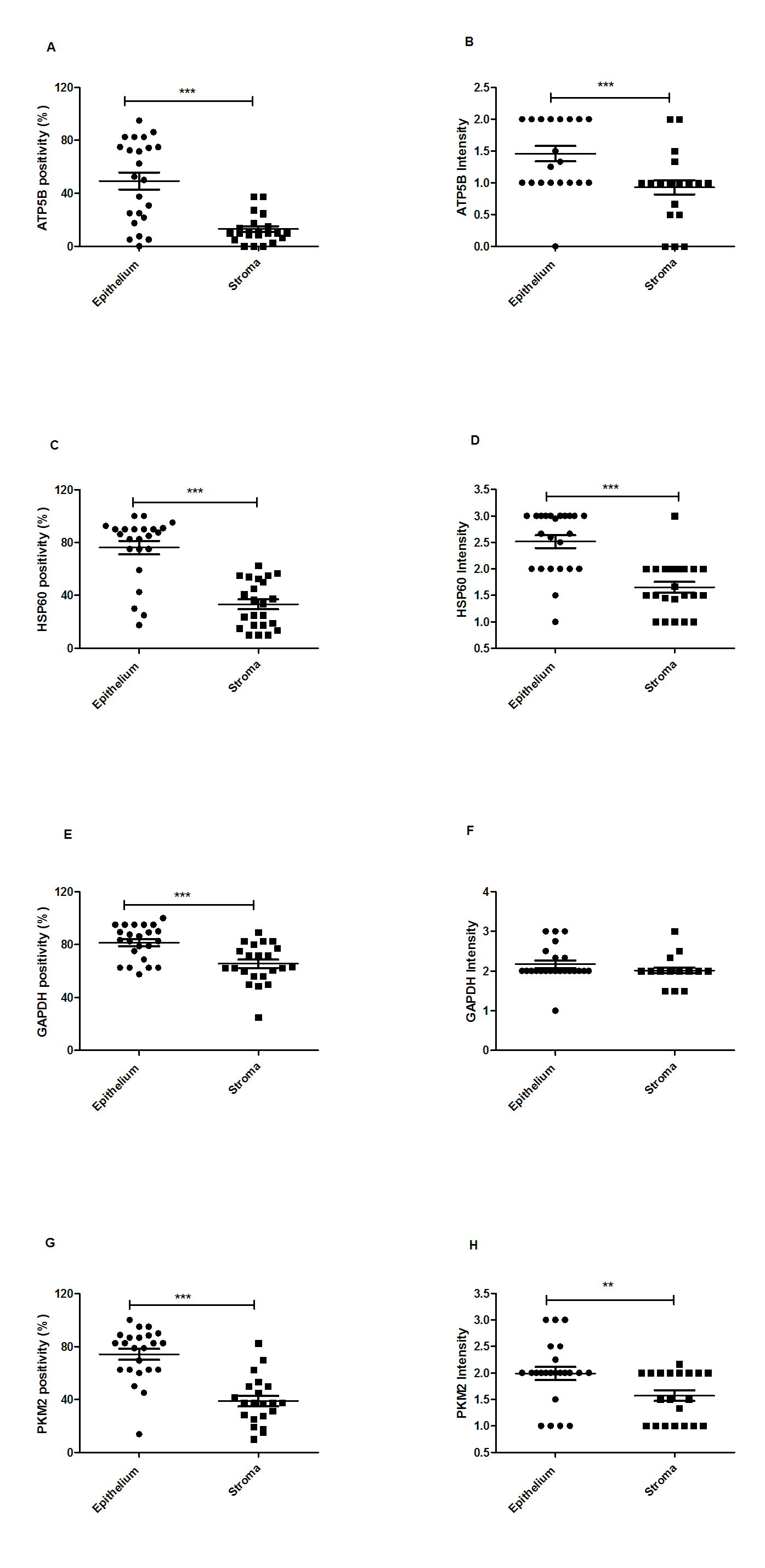

Supplement: Figure S3 — Metabolism is increased in the tumour epithelium in pre-treatment OAC tumours. Expression (percentage positivity and intensity) of ATP5B (A and B), HSP60 (C and D), GAPDH (E and F) and PKM2 (G and H) was assessed in the stromal and epithelial compartments in pre-treatment OAC biopsies (n = 23). Statistical analysis was performed by paired 2-tailed Student’s t-test or Wilcoxon non-parametric test. **P<0.01, ***P<0.001. (TIF) [file pone.0100738.s003.tif]
