# Supplementary material for: Altered Mitochondrial Function and Energy Metabolism Is Associated with a Radioresistant Phenotype in Oesophageal Adenocarcinoma
Source: PLoS One. 2014 Jun 26;9(6):e100738. doi: 10.1371/journal.pone.0100738 (PMC4072695; doi:10.1371/journal.pone.0100738)
Supplement: File S1 — (DOCX) [file pone.0100738.s004.docx]

**Supporting Materials and Methods**

**ATP5B qPCR**

Total RNA was reverse transcribed to cDNA using random hexamers (Invitrogen) and BioScript^TM^ (Bioline). qPCR was performed using a TaqMan® primer probes (Applied Biosystems) and 18S was used as an endogenous control for data normalisation. Data was analysed using SDS 2.3 and SDS RQ 1.2 relative quantification software (Applied Biosystems). One control sample was set as the calibrator for the analysis.

**Western blotting**

Cells were lysed in RIPA buffer (50 mM Tris, pH 7.5, 150 mM NaCl, 2 mM EDTA, pH 8.0, 0.5% Triton X-100, protease inhibitor cocktail, 1 mM PMSF and 1 μM Na_3_VO_4_). Sample protein concentrations were quantified using the BCA assay (Pierce). Proteins (30 μg) were resolved on a 12% polyacrylamide gel and transferred to polyvinylidene fluoride membranes. Immunoblots were incubated with mouse anti-human ATP5B antibody (Santa Cruz Biotechnology), 1:500 dilution or rabbit anti-human β-actin (Abcam), 1:2,000 dilution, followed by incubation with HRP-labelled donkey anti-mouse (R&D systems) or goat anti-rabbit (Santa Cruz Biotechnology) IgG antibodies, respectively. Detection was performed using SuperSignal West Pico chemiluminescent substrate kit (Thermo Scientific). Densitometric analysis was performed using ImageJ software.

**Supporting Figure Legends**

**Figure S1. Immunohistochemical staining in pre-treatment OAC tumours.** Stromal expression of (**A)** ATP5B, (**C)** HSP60, (**E)** GAPDH, (**G)** PKM2 and epithelial expression of (**B)** HSP60, (**D)** GAPDH, (**F)** PKM2 was assessed in pre-treatment OAC biopsies from patients who subsequently had a good (*n* = 7) or poor (*n* = 16) response to neoadjuvant CRT.

**Figure S2. ATP5B expression in OE33 P and OE33 R.** ATP5B expression was assessed in OE33 P and OE33 R, basally and at 6 h and 24 h post irradiation with 2 Gy by **(A)** qPCR and **(B)** western blotting. Data are presented as the mean ± SEM from 3 independent experiments. **(C)** Representative western blot.

**Figure S3. Metabolism is increased in the tumour epithelium in pre-treatment OAC tumours.** Expression (percentage positivity and intensity) of ATP5B (**A and B**), HSP60 (**C and D**), GAPDH (**E and F**) and PKM2 (**G and H**) was assessed in the stromal and epithelial compartments in pre-treatment OAC biopsies (*n* = 23). Statistical analysis was performed by paired 2-tailed Student’s *t*-test or Wilcoxon non-parametric test. ***P* < 0.01, *** *P* < 0.001.
